# Evaluation of the Antimicrobial Properties and Shear Bond Strength of Conventional Orthodontic Adhesive Modified by Green-Synthesized Titanium Dioxide Nanoparticles Admixed With Calotropis gigantea Flowers: An In Vitro Study

**DOI:** 10.7759/cureus.96386

**Published:** 2025-11-08

**Authors:** Yarasi Himaja, Embeti Srikanth, Madiraju Sirisha Rao, Divya Ravuru, JS Yamini Priyanka, Prasad Mandava, Gowri Sankar Singaraju

**Affiliations:** 1 Orthodontics and Dentofacial Orthopedics, Narayana Dental College, Nellore, IND; 2 Public Health Dentistry, CKS Theja Institute of Dental Sciences and Research, Tirupati, IND; 3 Orthodontics and Dentofacial Orthopedics, Oxford Dental College, Bengaluru, IND; 4 Orthodontics and Dentofacial Orthopedics, Vydehi Institute of Dental Sciences, Bengaluru, IND

**Keywords:** adhesive, calotropis gigantea, green synthesis, nanoparticles, orthodontics, shear bond strength, titanium dioxide

## Abstract

Background

Titanium dioxide (TiO₂) nanoparticles (NPs) have been incorporated into orthodontic adhesives to enhance antibacterial performance. However, limited data exist on their biologically synthesized forms derived from herbal sources.

Objective

This study aimed to evaluate the antimicrobial activity and shear bond strength (SBS) of a conventional orthodontic adhesive modified with green-synthesized (GS) TiO₂ nanoparticles prepared using *Calotropis gigantea* flower extract.

Methods

Forty premolars extracted for orthodontic reasons were randomly allocated to five groups (n = 8). Group I served as control (unmodified adhesive). Experimental groups were as follows: Group II, adhesive + 1% TiO₂ NPs; Group III, adhesive + 5% TiO₂ NPs; Group IV, adhesive + 1% GS-TiO₂ NPs; and Group V, adhesive + 5% GS-TiO₂ NPs. Green synthesis employed ethanolic extracts of dried *C. gigantea* flowers as reducing and stabilizing agents. Nanoparticle formation was confirmed by color change and characterized by Fourier-transform infrared spectroscopy (FTIR), scanning electron microscopy (SEM), ultraviolet (UV)-visible spectrophotometry, energy-dispersive X-ray spectroscopy (EDS), particle-size analysis, and zeta potential measurement. Antimicrobial activity was tested using agar well and disc agar diffusion (DAD) assays, and SBS was measured with a universal testing machine.

Results

The incorporation of TiO₂ NPs reduced microbial growth compared to the control adhesive. The mean SBS for the control (Group I) was 14.79 ± 1.51 MPa. SBS decreased with increasing NP concentration: Group II = 13.66 ± 1.63 MPa, Group III = 10.33 ± 1.08 MPa, Group IV = 9.09 ± 0.92 MPa, and Group V = 8.15 ± 0.99 MPa (p < 0.001).

Conclusions

Both antimicrobial activity and SBS declined with higher TiO₂ concentrations, and the green-synthesized formulations showed slightly lower values than conventionally synthesized TiO₂ adhesives. Nevertheless, all SBS values remained within the clinically acceptable range for orthodontic bonding.

## Introduction

Orthodontic treatment with fixed appliances inevitably accumulates dental plaque around brackets and archwires, resulting in bacterial overgrowth in patients' saliva and dental plaque, notably *Streptococcus mutans *(SM) and *Lactobacillus*. Researchers have identified these microorganisms as the primary pathogens in dental caries, and their presence heightens the risk of decalcification, leading to incipient caries or white-spot lesions (WSLs) [1,2]. The first step in preventing WSL is maintaining excellent oral hygiene to prevent plaque formation; however, it relies entirely on patient compliance [3]. Poor patient compliance is a well-documented issue, with less than 15% of orthodontic patients maintaining hygiene as instructed [4]. To address this problem, researchers are exploring new methods to make orthodontic materials, such as adhesives, ligatures, and brackets, that have inherent antibacterial or anti-cariogenic properties [4].

The orthodontic adhesive is in intimate contact with the enamel surface, and different methods have been tried with adhesives to provide antibacterial properties [1,4,5-8]. Nanotechnology facilitated the development of "experimental composite adhesives" utilizing nanoparticles (NPs), which offer enhanced antibacterial activity [9]. Particles that are insoluble and less than 100 nm in size are considered nanoparticles (NPs) [10]. When compared to non-nanoscale particles, these NPs have a high surface-to-volume ratio. This makes it easier for them to interact with the membranes of bacterial cells and offers them a lot more surface area to kill bacteria. While several bacterial strains are developing resistance to antibiotics, the development of resistance against metal nanoparticles is likely to be less pronounced than that against conventional antibiotics. These factors have promoted alternative antibacterial agents, such as metallic NPs [11].

Previous studies have assessed the antimicrobial effectiveness of different metal NPs and organic extracts incorporated into orthodontic composite resins, all showing promising results [3,6,7,12-15]. The research by Khashan et al. definitively establishes that nano-titanium dioxide (TiO₂) possesses significant antibacterial properties and can be utilized effectively as an antimicrobial agent across various applications [15]. The use of synthetic materials (metal NPs) causes the release of metal ions, which can exhibit cytotoxicity risks [6]. Finding natural products with antiplaque and antimicrobial properties that replace the metal ions completely or partially could be highly beneficial. Green synthesis (GS), a technique that offers environmentally friendly alternatives, has seen an increase in the utilization of natural products and active plant extracts in synthesizing NPs [9]. Recently, *Calotropis gigantea*, a medicinal plant, and its extracts have attracted researchers' attention. It is a member of the "Asclepiadaceae" family and grows naturally in Asian countries. It is rich in bioactive compounds and has gained attention for its diverse medicinal and antimicrobial properties [16-18].

Few studies have established the antibacterial efficacy of the *C. gigantea* leaf extract in vitro [19] and in vivo studies using mouth rinses in the dental field [20]. Currently, only one study has been conducted on orthodontic adhesives using *C. gigantea* leaf extract NPs, which has shown promising outcomes [21]. The fresh flowers of *C. gigantea* have been shown to be effective against *Streptococcus mutans* and other pathogens that cause dental caries [21]. No orthodontic study has used *C. gigantea* flowers in the literature for the green synthesis of titanium dioxide nanoparticles. We undertook this study with the premise that admixing green-synthesized TiO₂ nanoparticles with a conventional orthodontic adhesive could serve a dual purpose: enhancing the bond strength and improving the adhesive's anti-cariogenic potential. This approach was expected to mitigate the cytotoxicity concerns associated with purely metallic nanoparticles reported in earlier studies. The primary objective was to evaluate the shear bond strength (SBS) of adhesives modified with green-synthesized TiO₂ compared to conventionally synthesized TiO₂ and the unmodified control. The secondary objective was to assess the antimicrobial efficacy of these formulations against *Streptococcus mutans* and *Lactobacillus acidophilus *(LA).

It was hypothesized that the incorporation of green-synthesized TiO₂ would not significantly reduce SBS or antimicrobial activity relative to the non-admixed adhesive, remaining within clinically acceptable bond strength limits.

## Materials and methods

**Figure 1:**
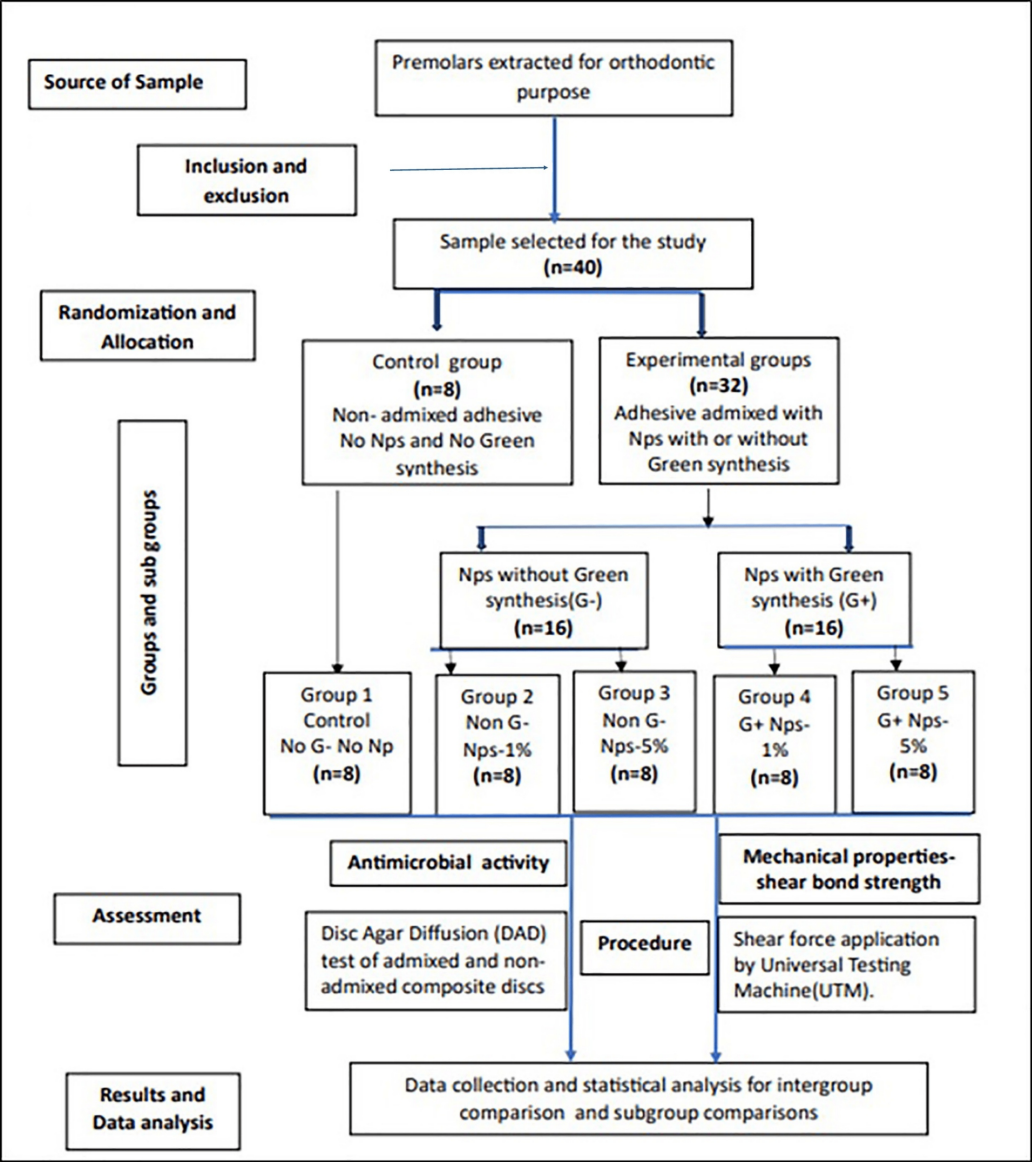
Study flowchart depicting sample selection, randomization, subgroup allocation, and assessment procedures. NPs: nanoparticles

The present study was conducted in the Department of Orthodontics and Dentofacial Orthopedics, Narayana Dental College and Hospital, Nellore, India, in collaboration with Dextrose Technologies Pvt. Ltd. (Bangalore, India) and Virtue Meta-Sol Laboratories (Hyderabad, India). The Institutional Ethical Committee of Narayana Dental College reviewed and approved the study protocol (approval number: D210040812). The overall study design, sample allocation, and testing sequence are summarized in Figure 1.

Sample size estimation

The sample size was calculated using G*Power software version 3.1.9.6 (Heinrich-Heine-Universität Düsseldorf, Düsseldorf, Germany). A minimum of 30 samples was required to detect a 1 mm difference in the antibacterial inhibition zone and a 1 MPa difference in shear bond strength (SBS) between any two samples, with an 80% power, a 5% alpha error, and a 95% confidence interval. Three additional samples were included in each group to account for potential loss, resulting in a total of 40 samples divided equally among the groups (n = 8 per group).

Collection of the sample

Forty maxillary and mandibular premolar teeth, which were extracted for orthodontic purposes, were collected and stored in a 0.1% thymol solution. Human premolar teeth indicated for orthodontic extraction were included, while those with caries, restorations, endodontic treatment, enamel hypoplasia, or enamel cracks were excluded.

The total sample consisted of 40 specimens, which were randomly allocated into five experimental groups, with eight samples in each group. Group I (A - control) included the unmodified traditional adhesive and served as the control. Group II (A + 1% TiO₂) comprised adhesive specimens containing 1% titanium dioxide (TiO₂) nanoparticles synthesized without green synthesis. Group III (A + 5% TiO₂) included adhesive samples incorporating 5% TiO₂ nanoparticles, also prepared without green synthesis. Group IV (A + 1% GS-TiO₂) represented adhesive specimens containing 1% TiO₂ nanoparticles obtained through green synthesis (GS). Finally, Group V (A + 5% GS-TiO₂) consisted of adhesive specimens with 5% TiO₂ nanoparticles produced via green synthesis.

Procedure

**Figure 2:**
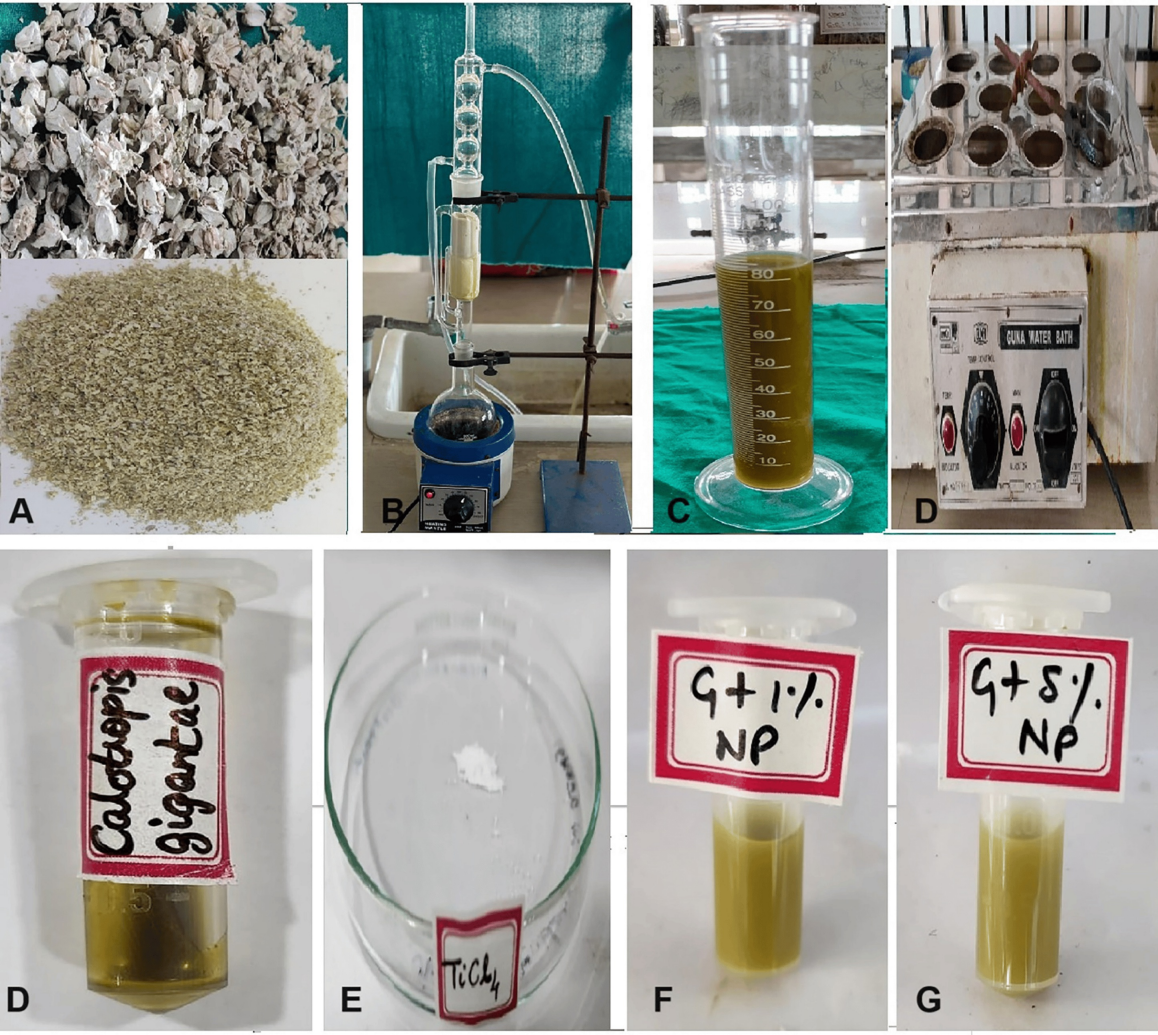
Steps involved in the preparation of green-synthesized (GS) TiO₂ nanoparticles (NPs) using Calotropis gigantea flower extract. (A) Dried flowers of *C. gigantea* and their powdered form. (B) Soxhlet extraction setup showing the solvent extraction of the powdered flowers. (C) Collected extract after the completion of the extraction process. (D, upper) Solvent evaporation using a water bath maintained at 60°C to obtain the crude extract. (D, lower) The obtained *C. gigantea* crude extract stored in individual labeled tubes for nanoparticle synthesis. (E) Titanium tetrachloride (TiCl₄) used as the precursor. (F) Green-synthesized TiO₂ nanoparticles prepared with 1% extract concentration (GS + 1% NP). (G) Green-synthesized TiO₂ nanoparticles prepared with 5% extract concentration (GS + 5% NP). TiO₂: titanium dioxide

The methodology used in this study was adapted from previously published reports [1,10,19,21-24]. Fresh *C. gigantea* flowers were washed, dried, and then powdered. The powder was dissolved in ethanol using a Soxhlet apparatus at 60°C for six hours to obtain the ethanolic extract [19,24]. After solvent evaporation, researchers stored the crude flower extract in a desiccator for future use (Figure 2).

Green synthesis of TiO₂ nanoparticles

Green synthesis was performed using titanium tetrachloride (TiCl₄; CAS 7550-45-0, 99% purity, Nano Research Lab, Jamshedpur, India) as the titanium precursor, while ethanol acted as both the solvent and a mild hydrolysis medium [10]. TiCl₄ is a volatile liquid that readily reacts with ethanol and trace water to form intermediate titanium alkoxide and hydroxide species, which, upon drying, yield amorphous TiO₂. The prepared plant extract was added dropwise to the precursor solution while continuously stirring at a controlled temperature.

To obtain the desired nanoparticle concentrations, 0.01 g of green-synthesized TiO₂ powder was incorporated into 1 g of Transbond XT (3M Unitek, Monrovia, CA) adhesive to obtain the 1% sample, and 0.05 g was used for the 5% sample, following the standardized protocol of Ravuru et al. (2023) [21]. During green synthesis, the ethanolic extract of *C. gigantea* was added dropwise to the TiCl₄ precursor solution under continuous stirring until the color transition indicated nanoparticle formation. The synthesized particles were then filtered, washed with distilled water and ethanol, oven-dried, and calcined at 400°C-800°C to remove organic residues and promote crystallization. This controlled calcination preserves the morphological and phase characteristics imparted by plant biomolecules.

Characterization of GS-TiO₂ NPs

**Figure 3:**
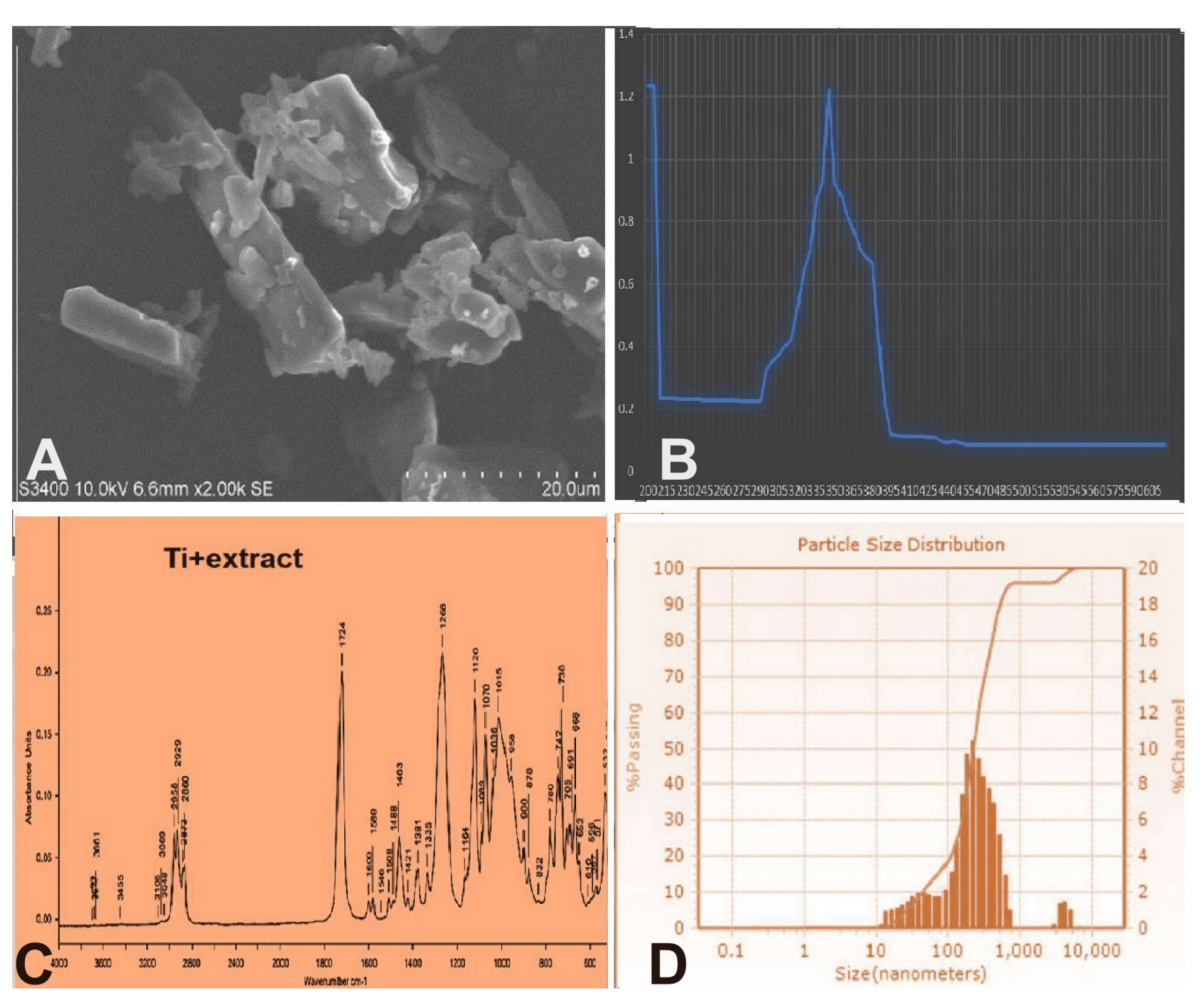
Characterization of green-synthesized titanium dioxide (TiO₂) nanoparticles (NPs). (A) Scanning electron microscopy (SEM) image revealing rod- and platelike nanoparticles measuring approximately 600-800 nm at 10 kV and 2,000× magnification. (B) Ultraviolet (UV) spectrophotometric profile showing a strong absorption peak characteristic of TiO₂ NPs, confirming optical bandgap excitation. (C) Fourier-transform infrared spectroscopy (FTIR) spectrum of the TiO₂ NPs prepared with *Calotropis gigantea* flower extract, indicating the presence of functional groups (O-H, C=O, Ti-O, and Ti-O-Ti) responsible for nanoparticle formation and stabilization. (D) Particle-size distribution curve demonstrating the nanoscale dispersion of the synthesized TiO₂ NPs, confirming uniform particle formation.

The formation of green-synthesized (GS) TiO₂ NPs was confirmed through multiple analytical techniques. Scanning electron microscopy (SEM, Zeiss EVO LS15, Bangalore, India), which operated at a 20 kV accelerating voltage and magnifications up to 20,000×, revealed irregularly agglomerated spherical clusters measuring 0.6-0.8 µm. Ultraviolet (UV)-visible spectrophotometry (LABMAN UV1900, Chennai, India) exhibited a distinct absorption peak at 345 nm corresponding to the Ti-O charge-transfer transition characteristic of anatase TiO₂. Fourier-transform infrared spectroscopy (FTIR, HP, Palo Alto, CA) confirmed the presence of Ti-O stretching vibrations and indicated the loss of organic functional groups after calcination. Particle-size analysis and zeta potential were measured using a Nanotrac Wave II (Microtrac, Hyderabad, India), confirming nanoscale dimensions and colloidal stability. It should be noted that the FTIR analysis of the plant extract alone could not be performed due to experimental constraints, which is acknowledged as a limitation of the study (Figure 3). 

Preparation of experimental composite adhesives

Conventional Transbond XT (3M Unitek, Monrovia, CA) served as the base material [1]. A 10% *C. gigantea* TiO₂ NP adhesive was prepared by manually mixing 300 mg of NP powder with 2,700 mg of base composite. This mixture was serially diluted with plain composite to yield 1% and 5% concentrations. The 1% and 5% GS-TiO₂ adhesives constituted Groups IV and V. Similarly, adhesives containing conventionally synthesized TiO₂ NPs were prepared for Groups II and III using the same protocol.

All mixtures were manually blended on a glass slab under dim light until uniform dispersion was achieved, measured using a digital precision scale.

Composite disc preparation

Light-cured composite discs (6 mm diameter × 1 mm thickness) were fabricated using standardized silicone molds. The discs were prepared from a conventional orthodontic adhesive (Transbond XT, 3M Unitek, Monrovia, CA) and polymerized with an LED curing unit in accordance with the manufacturer's instructions. These specimens were used for antimicrobial testing. The sample dimensions, curing protocol, handling procedures, and analytical parameters were adapted from a previous study, which evaluated nanoparticle-modified orthodontic adhesives under comparable laboratory conditions and assessed both antimicrobial activity and shear bond strength (SBS) [21].

Parameters tested

Antimicrobial Testing

The antibacterial activity of the isolated GS-TiO₂ nanoparticles was tested using the agar well diffusion method [1]. Ten milligrams of nanoparticles were dispersed in 2 mL of distilled water, and 50 µL aliquots were placed in wells on Mueller-Hinton agar plates inoculated with mixed bacterial culture. Plates were incubated at 37°C for 24 hours, and inhibition zones were measured.

**Figure 4:**
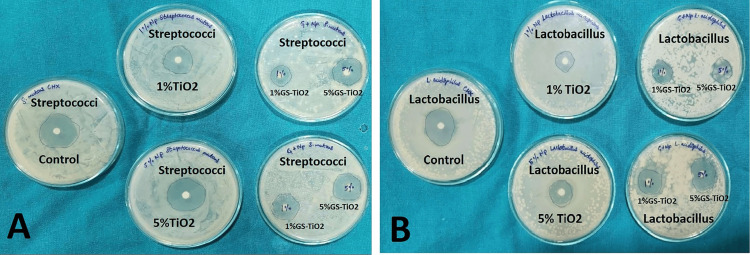
Antimicrobial activity of green-synthesized (GS) titanium dioxide nanoparticles (TiO₂ NPs) against Streptococcus mutans (A) and Lactobacillus acidophilus (B) using the disc agar diffusion test. Each Petri plate shows zones of inhibition produced by the control, 1% TiO₂, and 5% TiO₂ formulations, as well as the green-synthesized TiO₂ nanoparticles (1% GS-TiO₂ and 5% GS-TiO₂). The enhanced inhibitory zones around the GS-TiO₂ discs confirm the superior antibacterial efficacy of the green-synthesized nanoparticles compared to conventional TiO₂.

Composite discs were evaluated using the disc agar diffusion (DAD) test (Figure 4). *Streptococcus mutans* (ATCC 25175) and *L. acidophilus* (ATCC 4356) were reconstituted and cultured under anaerobic conditions at 37°C. Bacterial suspensions (10⁸ colony-forming units {CFU}/mL) were prepared spectrophotometrically, inoculated on Mueller-Hinton agar supplemented with 5% sheep blood, and composite discs were placed on the plates. After 24 hours of incubation at 37°C in CO₂, inhibition zones were measured using Vernier calipers.

Mechanical Testing: Shear Bond Strength

**Figure 5:**
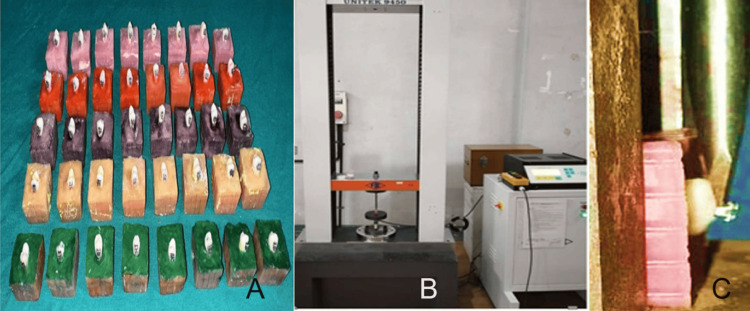
Shear bond strength (SBS) testing using the universal testing machine. (A) Premolar teeth embedded in color-coded acrylic resin blocks representing different experimental groups. (B) Mounted specimen positioned on the lower jaw of the universal testing machine (UNITEK UTM, model 9450, FIE, Kolhapur, India) for debonding evaluation. (C) Close-up (inset) showing the measurement of shear bond strength at the bracket-enamel interface using the bond failure method.

The extracted teeth assigned to each of the groups were embedded in colored acrylic blocks (1 × 1 × 1 inch) so that only the coronal portion of the specimen was visible, with the crown oriented along the long axis of the acrylic block (Figure 5). The buccal surfaces of all teeth were cleaned using a prophylaxis brush, rinsed with water, and then dried. The enamel was etched with 37% phosphoric acid (Prime-Dent® Etchant Gel, Prime Dental, Chicago, IL) for 30 seconds, then rinsed thoroughly, and air-dried using an oil-free air spray for 15 seconds. A thin layer of primer (Transbond XT Primer, 3M Unitek, Monrovia, CA) was applied to the buccal surface and light-cured for 10 seconds.

Metal brackets (Mini Diamond®, 0.022-inch slot, McLaughlin-Bennett-Trevisi {MBT} prescription, Ormco Corp., Brea, CA) were bonded to the center of the buccal surface using the adhesive corresponding to each group. Each bracket base had a surface area of approximately 12.62 mm². After the complete seating of the brackets with gentle pressure, excess adhesive was removed using a scaler, and the assembly was light-cured for 40 seconds (10 seconds from each aspect) using an LED curing unit (Woodpecker Light Cure LED Mini-S, 1,200 mW/cm², Equitech Engineers Pvt. Ltd., Pune, India). The bonding and curing parameters followed the standardized protocol previously reported by Ravuru et al. (2023) [21].

All bonded specimens were thermo-cycled in a thermocycling chamber (Thermotron S-8C, Holland, MI) for 1,000 cycles within 24 hours to simulate intraoral temperature fluctuations. Each cycle involved 15 seconds of immersion in a 5°C water bath, 10 seconds of dwell time, and 15 seconds of immersion in a 55°C water bath.

Following thermocycling, the shear force was applied to each bracket using an electronic universal testing machine (FIE UTES-40-HGF, Kolhapur, India), as shown in Figure 5. Specimens were mounted on the lower jaw of the unit connected to a 50 kg load cell. The bracket base was positioned parallel to the direction of the applied load, and shear force was applied at the bracket-adhesive interface using a 0.6 mm metal chisel blade in an occluso-cervical direction at a crosshead speed of 0.5 mm/minute until debonding occurred. The debonding force value (N) was divided by the bracket base area (mm²) to calculate the shear bond strength (SBS) in megapascals (MPa).

Data entry and statistical analysis

All data were compiled in Microsoft Excel (Microsoft Corp., Redmond, WA) and analyzed using DATAtab (version 2024, Graz, Austria). Normality was tested by the Shapiro-Wilk test. One-way ANOVA was used to compare groups, and pairwise differences were analyzed using the Bonferroni post hoc test. A p-value of <0.05 was considered statistically significant.

## Results

Characterization of the green-synthesized TiO₂ nanoparticles

The SEM analysis revealed that the prepared GS-TiO₂ nanoparticles exhibited irregularly agglomerated clusters with individual particle dimensions ranging from 600 nm to 800 nm at 10 kV accelerating voltage (Figure 3). UV-visible spectrophotometry demonstrated an absorption peak at 345 nm, a characteristic of Ti-O charge-transfer transitions, confirming TiO₂ formation. The FTIR spectrum showed a prominent peak at 1724 cm⁻¹, indicating the presence of organic functional groups likely originating from residual phytochemicals. Particle-size analysis revealed a heterogeneous distribution of TiO₂ plus extract particles ranging from 4 nm to 218 nm, confirming nanoscale formation as shown in Figure 3.

Zeta potential analysis displayed a positive surface charge, with a measured zeta potential of +16.4 mV, charge of 0.025 fC, and a field strength of 2.2 kV/m, signifying a moderately stable colloidal dispersion with high electrostatic repulsion. Together, these findings confirm the successful formation of stable, positively charged, green-synthesized TiO₂ nanoparticles.

The descriptive analysis of quantitative parameters, including microbial inhibition zone diameters for *Streptococcus mutans* and *Lactobacillus acidophilus*, as well as shear bond strength (SBS), is presented in Table 1. The normality of data was evaluated using the Shapiro-Wilk test for all quantitative variables. All parameters demonstrated p > 0.05, indicating that data for each group followed a normal distribution. Accordingly, parametric tests (one-way ANOVA, followed by Tukey's post hoc analysis) were applied for subsequent inter- and intragroup comparisons.

**Table 1:** Descriptive statistics and Shapiro-Wilk (SW) normality test results for all parameters across experimental groups. All parameters showed p > 0.05, indicating normal data distribution. GS-TiO₂, green-synthesized titanium dioxide; SD, standard deviation; CI, confidence interval

Parameter	Group	Mean ± SD	Median	Minimum	Maximum	95% CI of Mean	SW Value	P-value	Normality
*Streptococcus mutans* inhibition zone (mm)	G1 (control)	25.12 ± 0.68	25.26	24.10	26.20	24.6-25.6	0.95	0.612	Normal
	G2 (1% TiO₂)	15.84 ± 0.63	15.90	14.90	16.70	15.3-16.3	0.93	0.487	Normal
	G3 (5% TiO₂)	18.36 ± 0.54	18.40	17.50	19.10	17.9-18.8	0.91	0.352	Normal
	G4 (1% GS-TiO₂)	17.42 ± 1.81	17.60	14.50	19.30	16.2-18.6	0.88	0.214	Normal
	G5 (5% GS-TiO₂)	19.12 ± 1.68	19.00	16.90	21.20	18.0-20.2	0.89	0.265	Normal
*Lactobacillus acidophilus* inhibition zone (mm)	G1 (control)	24.25 ± 0.63	24.42	23.08	24.98	23.8-24.7	0.94	0.57	Normal
	G2 (1% TiO₂)	14.21 ± 0.57	14.26	13.09	14.92	13.8-14.6	0.94	0.624	Normal
	G3 (5% TiO₂)	17.92 ± 0.55	17.89	17.30	18.83	17.5-18.3	0.92	0.433	Normal
	G4 (1% GS-TiO₂)	16.98 ± 1.82	17.13	14.28	18.93	15.7-18.2	0.86	0.128	Normal
	G5 (5% GS-TiO₂)	18.64 ± 1.70	18.48	16.53	20.82	17.5-19.8	0.91	0.328	Normal
Shear bond strength (MPa)	G1 (control)	14.79 ± 1.51	14.30	12.60	17.40	13.7-15.8	0.96	0.772	Normal
	G2 (1% TiO₂)	13.66 ± 1.63	13.30	11.20	16.40	12.5-14.8	0.95	0.761	Normal
	G3 (5% TiO₂)	10.33 ± 1.08	10.20	8.60	11.80	9.6-11.1	0.96	0.825	Normal
	G4 (1% GS-TiO₂)	9.09 ± 0.92	8.75	7.90	10.60	8.5-9.7	0.93	0.545	Normal

Antimicrobial evaluation

The agar well diffusion assay demonstrated the complete inhibition of microbial growth in plates treated with the isolated GS-TiO₂ nanoparticles, indicating significant antibacterial activity. In the disc agar diffusion (DAD) test, composite discs containing TiO₂ nanoparticles exhibited clear inhibition zones against *S. mutans* and *L. acidophilus*, with statistically significant differences among groups (p < 0.001) (Table 2).

**Table 2:** Microbial zone of inhibition (mm) and shear bond strength (SBS) in megapascals of the groups evaluated. *Significant differences (p-value of <0.05). NP, nanoparticles; CI, confidence interval; DF, degree of freedom between groups; df, degree of freedom within groups; GS, green synthesis; TiO₂, titanium dioxide

Property	Group (NP%)	n	Mean	SD	CI	DF/df	F-value	P-value
Ia. Microbial zone of inhibition (mm): *Streptococcus mutans*	Group I (control)	8	26.77	0.59	26.36- 27.18	4.35	277.05	<0.001*
	Group II (1% TiO₂)	8	21.42	0.95	20.76-22.08			
	Group III (5% TiO₂)	8	25.62	0.7	25.13-26.1			
	Group IV (1% GS + TiO₂)	8	15.21	1.09	14.45-15.96			
	Group V (5% GS + TiO₂)	8	20.99	0.3	20.75-21.16			
Ib. Microbial zone of inhibition (mm): *Lactobacillus acidophilus*	Group I (control)	8	24.25	0.63	23.82-24.69	4.35	74.96	<0.001*
	Group II (1% TiO₂)	8	14.21	0.57	13.82-14.6			
	Group III (5% TiO₂​​​​​​​)	8	17.92	0.55	17.54-18.3			
	Group IV (1% GS + TiO₂​​​​​​​)	8	16.98	1.82	15.72-18.24			
	Group V (5% TiO₂​​​​​​​)	8	18.64	1.7	17.47-19.82			
II. Shear bond strength in MPa	Group I (control)	8	14.79	1.51	13.74-15.83	4.35	42.17	<0.001*
	Group II (1% TiO₂​​​​​​​)	8	13.66	1.63	12.53-14.79			
	Group III (5% TiO₂​​​​​​​)	8	10.33	1.08	9.57-11.08			
	Group IV (1% GS + TiO₂​​​​​​​)	8	9.09	0.92	8.45-9.73			
	Group V (5% GS + TiO₂​​​​​​​)	8	8.15	0.99	7.46-8.84			

For *S. mutans*, intrapair comparisons revealed statistically significant differences (p < 0.001) among all group combinations except G1-SM versus G3-SM and G2-SM versus G5-SM (Table 3).

**Table 3:** Pairwise comparison of S. mutans (SM) inhibition zone diameter (mm) of five groups: Bonferroni post hoc test. *Significant differences (p-value of <0.05). G1, control only adhesive without admixing; G2, adhesive admixed with 1% titanium; G3, adhesive admixed with 5% titanium; G4, adhesive admixed with 1% green synthesis + titanium dioxide nanoparticles; G5, adhesive admixed with 5% green synthesis + titanium dioxide nanoparticles; CI, confidence interval

Pairwise Comparison		Mean Difference	Standard Error	95% CI Lower Limit	95% CI Upper Limit	t	p
G1-SM	G2-SM	5.34	0.388	4.14	6.55	13.78	<0.001*
G1-SM	G3-SM	1.15	0.388	-0.06	2.36	2.96	0.055
G1-SM	G4-SM	11.56	0.388	10.35	12.77	29.8	<0.001*
G1-SM	G5-SM	5.81	0.388	4.6	7.02	14.98	<0.001*
G2-SM	G3-SM	-4.2	0.388	-5.41	-2.99	-10.82	<0.001*
G2-SM	G4-SM	6.22	0.388	5.01	7.42	16.02	<0.001*
G2-SM	G5-SM	0.46	0.388	-0.74	1.67	1.2	1
G3-SM	G4-SM	10.41	0.388	9.2	11.62	26.84	<0.001*
G3-SM	G5-SM	4.66	0.388	3.45	5.87	12.02	<0.001*
G4-SM	G5-SM	-5.75	0.388	-6.96	-4.54	-14.82	<0.001*

For *L. acidophilus*, statistically significant intrapair differences (p < 0.001) were observed among all pairs except G3-LA versus G4-LA and G3-LA versus G5-LA (Table 4).

**Table 4:** Pairwise comparison of L. acidophilus (LA) inhibition zone diameter (mm) of five groups: Bonferroni post hoc test. *Significant differences (p-value of <0.05). G1, control only adhesive without admixing; G2, adhesive admixed with 1% titanium; G3, adhesive admixed with 5% titanium; G4, adhesive admixed with 1% green synthesis + titanium dioxide nanoparticles; G5, adhesive admixed with 5% green synthesis + titanium dioxide nanoparticles; CI, confidence interval

Pairwise Comparison		Mean Difference	Standard Error	95% CI Lower Limit	95% CI Upper Limit	t	p
G1-LA	G2-LA	10.04	0.601	8.17	11.91	16.71	<0.001*
G1-LA	G3-LA	6.33	0.601	4.46	8.2	10.54	<0.001*
G1-LA	G4-LA	7.27	0.601	5.4	9.14	12.11	<0.001*
G1-LA	G5-LA	5.61	0.601	3.74	7.48	9.33	<0.001*
G2-LA	G3-LA	-3.71	0.601	-5.58	-1.84	-6.17	<0.001*
G2-LA	G4-LA	-2.77	0.601	-4.64	-0.9	-4.61	0.001*
G2-LA	G5-LA	-4.43	0.601	-6.3	-2.56	-7.38	<0.001*
G3-LA	G4-LA	0.94	0.601	-0.93	2.81	1.56	1
G3-LA	G5-LA	-0.73	0.601	-2.6	1.14	-1.21	1
G4-LA	G5-LA	-1.67	0.601	-3.54	0.2	-2.77	0.088

Shear bond strength (SBS) analysis

The SBS results demonstrated that the control group (Group I) exhibited the highest mean bond strength (14.79 ± 1.51 MPa), whereas the 5% GS-TiO₂ group (Group V) showed the lowest mean value (8.15 ± 0.99 MPa). A one-way ANOVA revealed a statistically significant difference in SBS among the five groups (p < 0.001) (Table 1). Pairwise comparisons indicated significant differences between most groups, except between G1:G2, G3:G4, G3:G5, and G4:G5 (Table 5).

**Table 5:** Pairwise comparison of shear bond strength (SBS) in megapascals of the five groups: Bonferroni post hoc test. *Significant differences (p-value of <0.05). G1, control only adhesive without admixing; G2, adhesive admixed with 1% titanium; G3, adhesive admixed with 5% titanium; G4, adhesive admixed with 1% green synthesis + titanium dioxide nanoparticles; G5, adhesive admixed with 5% green synthesis + titanium dioxide nanoparticles; CI, confidence interval

Pairwise Comparison		Mean difference	Standard Error	95% CI Lower Limit	95% CI Upper Limit	t	p
G1	G2	1.13	0.63	-0.84	3.09	1.79	0.828
G1	G3	4.46	0.63	2.5	6.42	7.08	<0.001*
G1	G4	5.7	0.63	3.74	7.66	9.05	<0.001*
G1	G5	6.64	0.63	4.68	8.6	10.54	<0.001*
G2	G3	3.34	0.63	1.38	5.3	5.3	<0.001*
G2	G4	4.57	0.63	2.61	6.54	7.26	<0.001*
G2	G5	5.51	0.63	3.55	7.47	8.75	<0.001*
G3	G4	1.24	0.63	-0.72	3.2	1.96	0.574
G3	G5	2.17	0.63	0.21	4.14	3.45	0.015
G4	G5	0.94	0.63	-1.02	2.9	1.49	1

## Discussion

Several methods to eliminate or reduce enamel demineralization or white-spot lesion (WSL) formation during orthodontic treatment mostly rely on patient compliance [3]. One effective method in preventing enamel demineralization, which is independent of patients' cooperation, is the use of orthodontic adhesives with antibacterial properties, making them resistant to the adhesion of bacteria and biofilm formation.

Various synthetic metallic NPs [1,24] or organically modified nanoparticles [13,14] were added to orthodontic composites and found to have antibacterial properties. Metallic NPs carry the risk of cytotoxicity [14]; therefore, using the NPs synthesized from natural products, especially plant extracts, would be beneficial. Green synthesis, also known as environmentally friendly and sustainable generation of nanoparticles, has become increasingly popular in biological processes due to its avoidance of harmful chemicals and toxic solvents. Green synthesis techniques, such as using plants and microbes, are natural sources commonly employed [19-22].

*Calotropis gigantea* is one such plant that is well known for its medicinal properties. Considering the current interest in the antimicrobial activity of the *C. gigantea* plant and the gap in evidence regarding its antimicrobial efficacy, we pursued this study using the flowers of this plant to assess its antimicrobial efficacy when used in nanoparticle form, admixed with orthodontic adhesive. A deep look into the literature revealed that only three studies have been conducted on this plant in the dental field, and no such studies have been conducted so far using this plant from the perspective of orthodontics [19-22].

Using the manual mixing method [1], *C. gigantea* TiO₂ nanocomposites of 1% and 5% concentrations were prepared, and antimicrobial activity was assessed against *S. mutans* and *L. acidophilus*, the key organisms in the initiation and progression of caries [1,2].

The agar well diffusion and DAD tests assessed antimicrobial activity. The agar well test showed that *C. gigantea* NPs could stop bacteria from growing, and the DAD test measured the diameters of the inhibition zones around the discs. The results showed that the adhesive could stop WSLs from forming at the bracket-enamel interface [1].

In this study, inhibition zones around the unmixed adhesives for both *S. mutans* and *L. acidophilus* were broader than those of the test groups. Increasing TiO₂ concentration was associated with larger inhibition zones for *S. mutans*. The 1% and 5% GS-TiO₂ groups showed smaller zones than the non-GS-TiO₂ adhesives. For *S. mutans*, inhibition ranged from 15.21 ± 1.09 mm (Group IV) to 26.77 ± 0.59 mm (control) and, for *L. acidophilus*, from 14.21 ± 0.57 mm (Group II) to 24.25 ± 0.63 mm (control).

In clinical settings, the exposed enamel margin around brackets is roughly 2-3 mm; thus, the inhibition diameter achieved with 1% *C. gigantea* NPs lies within a realistic antibacterial range. A comparable experiment using *C. gigantea* extract from leaves showed smaller inhibition zones (8-11 mm) [21]; the larger zones in the current study may stem from synergistic effects of the TiO₂ core with organic floral residues retained post-calcination.

A study using curcumin nanoparticles admixed with adhesives found no inhibition zones at 1%-10% concentrations after 48 hours of DAD tests, though biofilm assays showed long-term bacterial reduction. Pourhajibagher et al. [14] reported sustained antimicrobial activity of photoactivated cationic curcumin-doped zinc oxide nanoparticles (cCur/ZnO NPs) up to 60 days, whereas Sodagar et al. [13] observed propolis NPs effective only against *Streptococcus* species, not *Lactobacillus*. In our study, the surface treatment of TiO₂ NPs with *C. gigantea* slightly reduced diffusion capacity but maintained zones within clinical limits.

Mirhashemi et al. found that chitosan-coated ZnO NPs generated larger inhibition zones at elevated concentrations, whereas our control group displayed the most extensive zones [25]. Sodagar et al. reported 10-11 mm zones for 10% TiO₂ NPs, smaller than our 5% results [1]. Silver (Ag) and copper oxide NPs [24,26] also displayed concentration-dependent antibacterial effects, whereas some studies [7,13] found no zones for Ag NPs. Thus, microbial inhibition depends on NP type, concentration, surface chemistry, and exposure duration.

No previous study has compared GS-TiO₂ NPs with non-green-synthesized TiO₂. Differences in the *S. mutans* levels between the GS-TiO₂ and TiO₂ groups were not significant, but differences between the GS and non-GS groups were significant (p < 0.05). No consistent pattern emerged for *L. acidophilus*.

The SBS results showed that incorporating any additive reduced bond strength, with the unmodified adhesive the highest (14.79 ± 1.51 MPa) and 5% GS-TiO₂ the lowest (8.15 ± 0.99 MPa). These values remain clinically acceptable [6]. Poosti et al. reported similar findings (14.3 MPa for 1% TiO₂ versus 14.4 MPa for control) [6]. Our data align with prior reports, which indicate that the incorporation of antimicrobial agents alters bonding and reduces shear bond strength (SBS) [1,13,14,26]. Sodagar et al. found reductions of 47% (1% TiO₂) and 59% (5% TiO₂) versus 34.47 MPa for controls [1]. In our study, the decrease was milder: 7% for 1% and 29% for 5%. Excluding our earlier work, most evidence indicates that metallic nanoparticles (NPs) maintain higher shear bond strength (SBS) compared to organic NPs [22]. Some studies attempted dual-metal additions [5,26] without success, though Felemban and Ebrahim [27] reported increased SBS with zirconium dioxide (ZrO₂)-TiO₂ NPs at 0.5-1 wt%. Although mean bond strength decreased with increasing nanoparticle concentration, all recorded values remained within or near the clinically acceptable range of 5.9-7.8 MPa, allowing a 2-3 MPa safety margin for routine orthodontic bonding [28].

In the current study, the green synthesis of TiO₂ nanoparticles by utilizing *Calotropis gigantea* flowers was done by adding the plant extract dropwise into the titanium precursor solution and subjecting this to constant stirring [10]. The use of titanium tetrachloride (TiCl₄) and ethanol as a solvent classifies this process as a partially green or hybrid synthesis, because the plant extract served as a natural reducing and capping agent but not as the sole reagent. The subsequent calcination at 400°C-800°C was an essential step to eliminate residual organics and to achieve the crystalline anatase TiO₂ structure. This high-temperature step, although sometimes debated in fully biogenic protocols, is supported by studies such as those by Verma et al. [29] and Mulay et al. [30], which confirmed that calcination in the 700°C-800°C range improves purity, phase transformation, and optical properties of plant-mediated TiO₂ nanoparticles without destroying their biologically derived surface moieties.

We acknowledge that, unlike some previous phytosynthetic reports, the current study did not include a separate characterization of the crude *C. gigantea* flower extract (e.g., FTIR or UV-visible spectra of the extract alone). This omission limits the direct confirmation of the phytochemical functional groups responsible for reduction and capping. Nevertheless, the FTIR of the final GS-TiO₂ nanoparticles exhibited organic peaks around 1724 cm⁻¹, indicating that phytochemical residues persisted after calcination, consistent with partial green synthesis as described by Verma et al. [29] and Mulay et al. [30].

The darker shade of the NP-loaded adhesive and manual mixing may have reduced light penetration and polymerization depth, thereby influencing SBS. Minor discrepancies in reported particle sizes could be due to nanoparticle agglomeration and the differing measurement principles of dynamic light scattering (DLS) (primary particle size) and SEM (agglomerated clusters). In addition, dispersion uniformity, the degree of polymer conversion, and cytotoxicity assays were not performed and should be explored in future studies. The in vitro design cannot fully replicate the dynamic oral environment; long-term biofilm and biocompatibility evaluations are recommended to confirm clinical safety.

Despite these limitations, the study offers several noteworthy strengths. It is the first to directly compare green-synthesized and conventionally synthesized TiO₂ nanoparticles under identical experimental conditions. The combination of antimicrobial and mechanical testing, together with multimodal nanoparticle characterization, provides a comprehensive assessment of performance. The methodology followed the validated protocol of earlier studies, ensuring the reliability and reproducibility of the findings.

Green-synthesized TiO₂ nanoparticles derived from *Calotropis gigantea* flowers enhanced the antibacterial properties of orthodontic adhesives while maintaining clinically acceptable bond strength. This bioinspired modification can reduce bacterial colonization and minimize white-spot lesion formation around brackets without relying on patient compliance.

The material offers a sustainable, low-toxicity alternative to conventional metallic nanoparticles. Its 2-3 mm inhibition zone corresponds to the typical enamel margin around orthodontic brackets. Overall, GS-TiO₂-modified adhesives present a practical step toward preventive, biocompatible orthodontic bonding systems.

## Conclusions

The isolated *Calotropis gigantea*-derived TiO₂ nanoparticles exhibited distinct antimicrobial activity. When incorporated into orthodontic adhesive, the green-synthesized TiO₂ formulations showed slightly lower antibacterial efficacy than their conventionally synthesized counterparts, and both performed marginally below the non-admixed control yet retained bond strengths within clinically acceptable limits. The inverse relationship between nanoparticle concentration and antibacterial effect may be attributed to diffusion restrictions of densely packed particles, partial agglomeration within the resin matrix, or methodological variability. Although all nanoparticle-enriched adhesives exhibited reduced shear bond strength compared to the control, the values remained within the range considered adequate for orthodontic applications. Overall, low-concentration (1%) hybrid green-synthesized TiO₂ adhesives appear promising as biocompatible adjuncts for minimizing enamel demineralization during fixed-appliance therapy. Further studies evaluating nanoparticle dispersion, polymer-conversion degree, cytotoxicity, and long-term biofilm behavior are required before clinical translation.
